# Development and Evaluation of Novel Leflunomide SPION Bioemulsomes for the Intra-Articular Treatment of Arthritis

**DOI:** 10.3390/pharmaceutics14102005

**Published:** 2022-09-22

**Authors:** Haidy Abbas, Heba A. Gad, Nesrine S El Sayed, Laila Ahmed Rashed, Mohamed A. Khattab, Ahmad O. Noor, Mariam Zewail

**Affiliations:** 1 Department of Pharmaceutics, Faculty of Pharmacy, Damanhour University, Damanhour 22511, Egypt; 2 Department of Pharmaceutics and Industrial Pharmacy, Faculty of Pharmacy, Ain Shams University, Cairo 11566, Egypt; 3 Department of Pharmaceutical Sciences, Pharmacy Program, Batterjee Medical College, Jeddah 21442, Saudi Arabia; 4 Department of Pharmacology and Toxicology, Faculty of Pharmacy, Cairo University, Cairo 11562, Egypt; 5 Department of Medical Biochemistry and Molecular Biology, Faculty of Medicine, Cairo University, Cairo 11956, Egypt; 6 Department of Cytology and Histology, Faculty of Veterinary Medicine, Cairo University, Cairo 12211, Egypt; 7 Department of Pharmacy Practice, Faculty of Pharmacy, King Abdulaziz University, Jeddah 21589, Saudi Arabia

**Keywords:** rheumatoid arthritis, disease-modifying anti-rheumatics, stimuli responsive, local targeting, leflunomide, drug discovery, industrial development

## Abstract

Systemic treatments for rheumatoid arthritis are associated with many side effects. This study aimed to minimize the side effects associated with the systemic administration of leflunomide (LEF) by formulating LEF-loaded emulsomes (EMLs) for intra-articular administration. Additionally, EMLs were loaded with supramagnetic nanoparticles (SPIONs) to enhance joint localization, where a magnet was placed on the joint area after intra-articular administration. Full in vitro characterization, including colloidal characteristics, entrapment efficiency, and in vitro release were conducted besides the in vivo evaluation in rats with adjuvant-induced arthritis. In vivo study included joint diameter measurement, X-ray radiographic analysis, RT-PCR analysis, Western blotting, ELISA for inflammatory markers, and histopathological examination of dissected joints. The particle size and entrapment efficiency of the selected LEF SPION EMLs were 198.2 nm and 83.7%, respectively. The EMLs exhibited sustained release for 24 h. Moreover, in vivo evaluation revealed LEF SPION EMLs to be superior to the LEF suspension, likely due to the increase in LEF solubility by nanoencapsulation that improved the pharmacological effects and the use of SPION that ensured the localization of EMLs in the intra-articular cavity upon administration.

## 1. Introduction

Rheumatoid arthritis (RA) is a disease that attacks the joints all over the body and causes synovial cell proliferation and pannus formation. Pannus expansion causes cartilage and bone destruction, eventually disabling joints [1]. The exact cause of RA is unknown; however, there are many genetic and environmental factors that play a role in its development and progression [2]. There is growing evidence that RA has extra-articular manifestations that may extend to the cardiovascular system, skin, eyes, nervous system, lung, and kidney [3].

Systemic RA treatment protocols are associated with numerous gastrointestinal, renal, hepatic, and cardiac side effects [4]. Intra-articular (IA) injection is one of the approaches to reduce the side effects associated with administrating a drug systemically as they allow site-specific drug delivery, achieving high localized drug concentrations at the affected sites [5,6]. Nanotechnology is another approach to decrease the side effects of systemic administration by increasing the drugs bioavailability, in addition to their ability to achieve targeted drug delivery [7].

Superparamagnetic iron oxide nanoparticles (SPIONs) can direct the drug to the site of action with the aid of an outside magnetic field thus improving drug targeting [8]. They offer several advantages, including ease of preparation, biocompatibility, and the possibility of surface modification. In addition, SPIONs can be precisely and remotely controlled as they do not have a residual magnetic effect upon removal of the external magnetic field [9,10]. For magnetic drug targeting, the drug can be associated with SPION by direct attachment to the particles surface in different types of nanocarriers that hold the drug and SPION together [10,11,12,13,14,15].

On the other hand, over the years, RA treatment strategy has evolved from only controlling the symptoms with steroids and NSAIDs to suppressing RA progression with disease-modifying anti-rheumatics (DMARDs), biologics, and gene therapy [16]. One of the more efficient DMARDs is leflunomide (LEF), a prodrug. Upon administration, LEF is converted to teriflunomide, its active metabolite. It is thought to act by suppressing dihydroorotate dehydrogenase, which controls de novo pyrimidine biosynthesis [17], eventually regulating RA autoimmune reactions [18]. Moreover, it can inhibit TNFα-induced NF-κB activation [19] and suppress pro-inflammatory cytokines and prostaglandins production [20]. Furthermore, teriflunomide can inhibit osteoclastogenesis and osteoclast function, preventing bone loss and joint destruction [20,21]. Unfortunately, systemic LEF administration is associated with side effects, such as diarrhea, esophagitis, and colitis. Moreover, prolonged LEF administration may cause more serious side effects on the liver, lungs, and nerves [22,23,24].

The current study aimed to target LEF delivery to the joints intra-articularly. Thus, blank and LEF-loaded emulsomes (EMLs) were prepared and examined. The selected LEF-loaded EMLs formulation was loaded with SPION to enhance the EMLs joint retention. EMLs formulations were given to rats after induction of arthritis using antigen. Extensive in vitro and in vivo evaluations of the prepared formulations were conducted. To our knowledge, this is the first report of the preparation of LEF EMLs and LEF SPION EMLs and their application in the IA delivery for RA treatment.

## 2. Materials and Methods

LEF was purchased from Qingdao Franken Biochem Co. (Qingdao, China). Cholesterol was bought from Sigma-Aldrich (St. Louis, MO, USA). Lipoid^®^ S100 (l-α-phosphatidylcholine) and Compritol 888 ATO^®^ CA were supplied as a kind gift from Lipoid AG (Ludwigshafen, Germany) and Gattefossé (Lyon, France), respectively. All other chemicals and reagents that were used are of analytical grade.

### 2.1. Preparation of EMLs

EMLs were fabricated using the thin-film hydration method as reported by Rizk et al. [25]. Lipid phase consisting of Compritol, phosphatidylcholine, and cholesterol at a ratio of 1:1.2:0.4 (*w*/*w*) were dissolved in a sufficient amount of chloroform in a round-bottom flask. Then, chloroform was vaporized with the aid of low pressure at 45 °C using a rotary evaporator (N-1000; Tokyo Rikakikai Co., Ltd., Tokyo, Japan) until completely dry as evident by the appearance of a thin lipid film. To obtain EMLs, the formed film was hydrated with DW to yield a lipid concentration of 30 mg/mL. The hydration step was followed by ultrasonication (SonicaR 2200 EP S3, Soltec, Milan, Italy) for 20 min at 40% amplitude and further size reduction using the high-shear homogenization (Sonopuls HD 3100 ultrasonic homogenizer; Berlin, Germany).

The effects of various homogenization speeds between 10,000 and 15,000 rpm and different homogenization times between 0 and 15 min were studied. The obtained formulations were stored in the refrigerator till further use [26,27,28]. For LEF incorporation into the EMLs, LEF was dissolved in methanol, added to a mixture of lipids that had been dissolved in chloroform, and the same steps were followed to prepare EMLs as stated before [29]. The free unentrapped drug was filtered using Whatman^®^ filter paper No. 41 (20–25 µm pore size, Maidstone, UK) [6,30].

### 2.2. SPION Fabrication

SPION was prepared based on previous work [11]. In brief, ferric chloride hexahydrate (1.17 g) and ferrous sulfate tetrahydrate (0.6 g) were mixed in a molar ratio of 1.75:1 in 50 mL of DW with vigorous stirring until dissolved under N_2_ atmosphere at 70 °C. A 32% ammonium hydroxide was added to the mix with continuous stirring for one hour, and allowed to cool to 25 °C. This was followed by the separation of magnetite (Fe_3_O_4_) from the mixture with the aid of a magnet. Finally, SPION was washed five times using hot water and dried for 12 h in an oven at 50 °C.

### 2.3. Preparation of SPION-Loaded EMLs

SPION-loaded EMLs were formulated according to Abbas et al. [11]. First, 0.7 mL of 10.3 mg/mL of SPION aqueous dispersion was sonicated using a probe sonicator (Branson Sonifier^®^, Wilmington, NC, USA) for 10 s. Probe sonication of the formed coarse emulsion was continued for 1.5 min at 50% amplitude and 100 W. The probe sonication was stopped for 2 s intervals to avoid temperature rising. Next, the emulsion was allowed to cool in an ice bath for half an hour. The prepared formulae were subjected to freeze-drying (Freeze dryer Martin Christ, Alpha 1-2 LD; Vacuubrand and GMBH, Co Wertheim, Germany) for 48 h at −80 °C and 0.001 mbar and stored at 4 °C.

### 2.4. Evaluation of LEF-Loaded EMLs and LEF-SPION EMLs

#### 2.4.1. Particle Size Analysis and Zeta Potential Evaluation

The Zetasizer Nano ZS (Malvern Instruments, Worcestershire, UK) was used to measure the average particle size (PS), the size distribution (PDI), and the zeta potential (Z) of the EMLs. The scattering angle was 173 °C, and the temperature was 25 °C.

#### 2.4.2. Encapsulation Efficiency (EE)

Amounts of entrapped LEF within the EMLs were determined indirectly. In brief, a 1 mL aliquot of LEF EML suspension was centrifuged (Sigma Laboratory Refrigerated Centrifuge, Model 3K-3o, Osterode am Harz, Germany) at 4000 rpm at 4 °C for 1 h using a centrifuge tube fitted with an ultrafilter (Vivaspin 6VR, Sartorius, MWCO 10 kDa, Littleton, MA, USA). The amount of free LEF was measured spectrophotometrically at 263 nm [21,22,24], and EE % was quantified according to the following equation:

EE % = (Total amount of LEF − amount of unencapsulated LEF)/(Total amount of LEF) × 100(1)

#### 2.4.3. Transmission Electron Microscopy (TEM)

Morphological examination was carried out for the plain EMLs, LEF EMLs and LEF SPION EMLs (TEM: Jeol, JEM-1230, Tokyo, Japan). The EMLs were placed on a carbon-coated 300 copper grid followed by staining with 2% phosphotungstic acid for examination.

#### 2.4.4. Fourier Transform Infrared Spectroscopy Spectroscopic Analysis (FTIR)

The spectra of LEF, physical mixture of EMLs components and selected LEF loaded EMLs were analyzed at 500–4400 cm^−1^ using an Agilent Cary 630 Fourier transform infrared spectroscopy (FTIR) spectrometer (Santa Cala, CA, USA).

#### 2.4.5. Differential Scanning Calorimetry (DSC)

The thermal properties of the LEF loaded EMLs were explored using differential scanning calorimetry (DSC) (DSC Model 6, Perkin Elmer Instruments, Waltham, MA, USA) with a heating rate of 10 °C/min from 35 °C to 350 °C under nitrogen.

#### 2.4.6. In Vitro Drug and Kinetics Release

The dialysis bag technique was used to study the in vitro release of LEF from the EMLs in PBS pH 7.4 using in a water bath (Memmert GMBH, Schwabach, Germany) at 100 rpm and 37 ± 2 °C. An amount of EMLs corresponding to 500 µg of LEF was added to dialysis bags (cellulose membrane of MWT cut-off 12,000 g/mole, Sigma-Aldrich, St. Louis, MO, USA). At predetermined time intervals, an aliquot of 2 mL of the release medium was removed for analysis and instantly replenished with the same volume of fresh medium. The amount of LEF released was measured spectrophotometrically using first derivative of the UV spectrum at 248 nm [22,31]. Release data were fitted to zero-, first-order, and Higuchi and Korsmeyer–Peppas equations using DD solver 1.0 software (Microsoft Excel add in program).

#### 2.4.7. In Vivo Studies

##### Induction of RA and Experimental Design

**Figure 1:**
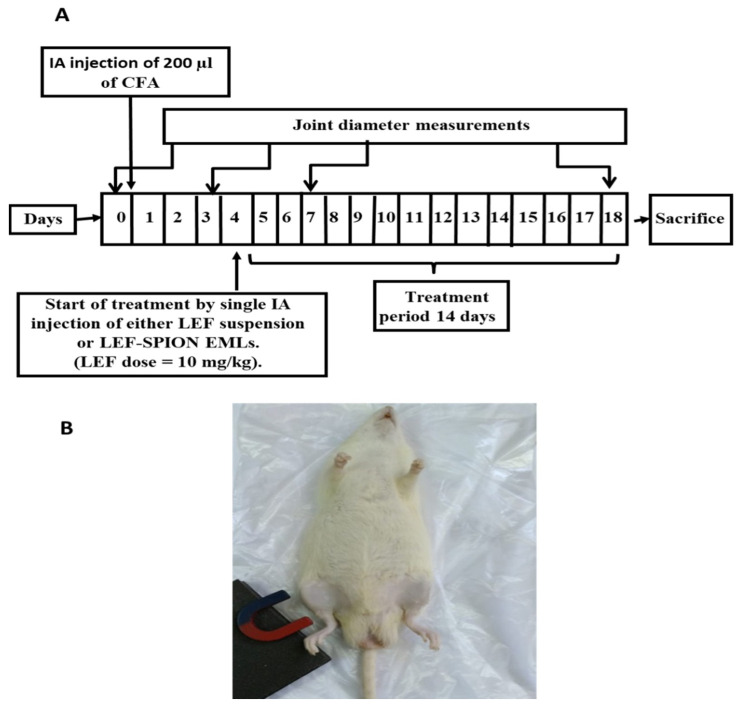
(**A**) Schematic representation of experimental design; (**B**) application of magnetic field following dose administration.

Adult male Sprague Dawley rats (150 ± 20 g) were kept in an air-conditioned room (25 ± 0.5 °C) with free access to a standard pellet diet and water under 12 h day/night cycles. The protocol for the study was approved by the Institutional Animal Ethics Committee of Faculty of Pharmacy, Cairo University, Egypt, and complies with the ARRIVE guidelines (Ethical approval number CU III 61-20). Thirty-six male rats were divided into four groups (n = 9 each). RA induction was carried out in the rats by injecting a 0.2 mL of complete Freund’s adjuvant (CFA) into the right knee joints; the left knee joints acted as the control. Rats were divided into four groups: the negative control, the positive control, the group receiving LEF-SPION EMLs, and the group receiving the LEF suspension. The administered LEF dose in LEF suspension or LEF SPION EMLs was equivalent to 10 mg/kg. The experimental design is illustrated in Figure 1A. For the group receiving LEF SPION EMLs, an external magnet was placed close to the rheumatic knee of a rat after IA injection (Figure 1B). After two weeks, rats were anesthetized, and blood samples were collected from retro orbital Venus plexus using a capillary tube and then centrifuged at 5000 rpm at ambient temperature for 15 min. The serum was frozen at −80 °C until analysis.

##### Measurement of Joint Diameter

The antero-posterior diameters of the rat knees were determined using a micrometer (KM-211-101, Shaanxi, China) at several time intervals (days 0, 4, and 7 and at the end of the treatment period). The aim was to monitor the effect of the treatments on the edema of rat knee joints. Measurements performed for each rat prior to the adjuvant injection were considered as the baseline value of the knee diameter.

##### X-ray Radiography

The radiographic examination was performed at the Surgery, Anesthesiology & Radiology Department, Faculty of Veterinary Medicine, Cairo University. All rats underwent general anesthesia using 2% Xylazine HCl at 5–10 mg/kg body weight and 5% Ketamine at 30–60 mg/kg body weight. The rats’ right knee joints were radiographed from the ventrodorsal (VD) view and mediolateral (ML) view using a FischerX-ray generating tube (Fischer R183, Emerald tube 125) with radiographic settings of 40 KV, 10 mA, and 80 cm FFD.

##### Quantitative Real-Time Polymerase Chain Reaction (qRT-PCR)

Analysis of PERK1/2, PJNK and p38-MAPK gene expression for total RNA extracted from rat dissected joints was carried out. First, RNA was extracted; then, 1 μg was reverse transcribed into complementary DNA using an RT-PCR kit (Stratagene, Cat. # 600188, La Jolla, CA, USA) according to the manufacturer’s instructions. Quantitative RT-PCR was accomplished using SYBR Green JumpStart Taq ReadyMix (Sigma–Aldrich, Cat. # S5193, St. Louis, MO, USA), where 5 μL of complementary DNA was added to 12.5 μL of SYBR Green, 5.5 μL of RNAse free water, and 2 μL of each primer (5 pmol/μL). The primer sequences are illustrated in Table 1. The mRNA levels of PERK 1/2, PJNK and P38 MAPK were normalized and presented as a ratio to β-Actin.

##### Western Blot Analysis

According to Zhou et al. [32], immunoblotting with a Western blot detection reagent (Amersham Biosciences in Piscataway, NJ, USA) was used to extract protein samples from rat knee articular cartilage to analyze phosphorylated cleaved caspase-3 and Bax/Bcl-2 ratio. The levels of the target proteins were normalized to that of β-Actin.

##### Enzyme-Linked Immunosorbent Assay (ELISA)

The serum levels of tumor necrosis factor-alpha (TNF-α), interleukin 1B (IL1B), nuclear factor (erythroid-derived)-Like 2 (NRF2), superoxide dismutase (SOD), and matrix metalloproteinases 9 (MMP9) were determined using an ELISA kit based on the stated instructions (Glory Science Co, Ltd., Del Rio, TX, USA).

##### Histopathologic Studies and Immunohistochemical Staining

Knee joint samples were fixed in 10% neutral buffered formalin for 48 h. The decalcification process was carried out using Cal-X II (Fisher Scientific, Waltham, MA, USA) for 10 days, samples processing using serial grades of ethanol and cleared in xylene followed by infiltration and embedding in paraplast tissue embedding media. Tissue sections of 5 microns were made by rotatory microtome at mid sagittal plane levels of all samples and then mounted on glass slides for H&E staining for general histological examination of tissue samples and Alcian blue pH 2.5 staining for assessment of cartilage matrix and proteoglycans staining [33,34]. Morphological assessment of tibiofemoral articular cartilage was conducted according to a modified Mankin scoring system [33,34,35] with 0 indicating normal cartilage and 13 indicating the maximal score of osteoarthritis. 

According to manufactures directions, deparaffinized antigen retrieved 5 μm thick tissue sections were treated with 3% H_2_O_2_ for 20 min, incubated with anti p-NFκB p65 antibody (GTX54672, GeneTex Inc.—1:100) overnight at 4 °C for 30 min, washed using immune washing Tris buffer, then incubated with secondary antibody HRP Envision kit (DAKO) 20 min, washed using immune washing Tris buffer, followed by diaminobenzidine (DAB) for 10 min, washed and then counter stained with hematoxylin, dehydrated and cleared in xylene and then cover slipped. Six random non-overlapping fields from synovial membrane of each sample were analyzed for immunoexpression levels of P-NFkB. Six random non-overlapping fields from articular cartilage of each sample were analyzed for mean reactive area percentage of proteoglycans in alcian blue stained tissue sections. All data were obtained by Leica Application module attached to Full HD microscopic imaging system (Leica Microsystems GmbH, Wetzlar, Germany).

### 2.5. Statistical Analysis

Results were presented as mean ± SD. Statistical analysis of the results was performed using Prism 7 software. For joint diameter measurements TWO WAY ANOVA followed by Tukey’s test with (*p* < 0.0001) was utilized. Student *t*-test with (*p* < 0.05) was applied for the analysis of colloidal EMLs characteristics and release data. One-way ANOVA followed by Tukey’s test with (*p* < 0.0001) was applied for the rest of the experimental results.

## 3. Results and Discussion

### 3.1. Characterization of LEF-Loaded EMLs and LEF SPION EMLs

The EMLs were selected to be loaded with LEF based on several features of EMLs. They are biocompatible nanosystems composed of a solid fat core enveloped by phospholipid layers and stabilized by cholesterol and soya lecithin. They combine the characteristics of emulsions and liposomes and are prepared using film hydration method like liposomes [28,36,37,38]. Therefore, they can increase the solubility and EE % and prolong the release of hydrophobic drugs [38]. Moreover, EMLs are easily formulated without any surfactants or cosurfactants; which makes them a secure choice over solid lipid nanoparticles and nanoemulsions. Furthermore, EMLs are more stable and can hold more drugs than liposomes and solid lipid nanoparticles [39,40]. Butoescu et al. reported that the microparticles loaded with dexamethasone acetate along with SPIONs have an excellent biocompatibility with synoviocytes and that they are internalized through a phagocytic process, as demonstrated by fluorescence-activated cell sorting and morphological analyses of cells exposed to microparticles. Histological analysis showed that the prepared microparticles did not induce any inflammatory reaction in the joint. This type of carrier could represent a suitable magnetically retainable intra-articular drug delivery system for treating arthritis [41].

To our knowledge, the preparation of LEF EMLs and LEF SPION EMLs has not been reported.

On the other hand, Compritol was chosen based on its hydrophobicity as it is composed of long-chain fatty acids [42]. Moreover, Compritol had been used in many studies for the fabrication of lipid nanocarriers for intraarticular drug injection for the treatment of arthritis [43,44,45,46]. In addition, cholesterol was included in the preparation to enhance the stabilization of the outer phospholipid layer besides being capable to augment loading and entrapment of hydrophobic drugs [25].

EMLs were fabricated by film hydration method using a minimum amount of organic solvents that were completely evaporated using rotary evaporator—the same method was previously used for the preparation of drug loaded liposomes for intra-articular delivery [47,48,49]. The effect of different homogenization times, speeds, and drug loading on the colloidal characteristics of EMLs was examined. Table 2 shows the compositions of the prepared EMLs. EMLs produced at a high homogenization speed of 15,000 rpm for 5 min (F5) had the smallest PS of 185.6 nm compared to other formulations produced at 10,000 rpm. As observed, increasing the homogenization time from 5 to 15 min at 10,000 rpm significantly decreased the PS (Student’s *t*-test; *p* < 0.05). The PSs of the F2, F3, and F4 preparations were 398.25, 365.3, and 223.6 nm, respectively. Moreover, increasing the homogenization speed decreased the EML PSs significantly. For example, the PS of F2 (prepared at 10,000 rpm) and F5 (15,000 rpm) were 398.25 and 185.6 nm, respectively. Our results are in line with Priyanka et al. [50], where the PS of Ficus religiosa L. extract loaded solid lipid nanoparticles decreases when the homogenization time and speed were increased. In contrast, our findings disagree with the results observed by Rizk et al. [25], who outlined that increasing the homogenization time corresponded to an increase in EML PS due to the increase in the total energy of the system [25]. Furthermore, LEF-loaded EMLs displayed a significant increase in PS compared with the blank formulations (Table 2). For example, the PS of F2, the blank preparation was 398.25 nm; however, upon LEF loading, the PS increased to 425.6 nm. Lastly, the PDI of all formulations was less than 0.3, indicating homogeneity of EML dispersion [51].

In order to prevent nanosystems aggregation, the zeta potential values are of great importance, as zeta potential values of more than 30 mV are thought to achieve sufficient nanosystems [10,52]. Here, all the prepared EML formulations showed a negative zeta potential value that was slightly higher in the LEF-loaded EMLs (Table 2).

The EE % of different EMLs formulations ranged from 74.2 (F2-LEF) to 87.3% (F5-LEF), likely attributed to the ability of the EMLs to increase the solubility and EE % of hydrophobic drugs [25,36] due to the highly hydrophobic Compritol and the presence of cholesterol [25].

F5-LEF showed the smallest PS and highest EE % with a low PDI value and was chosen to be loaded with SPION. The choice of SPION as magnetic material was based on their biocompatibility with joint tissues, especially with the synovial membrane, according to Schulze et al. [53]. Therefore, SPION is not expected to induce inflammatory or immunological reactions [54]. Here, incorporating SPION in EMLs slightly increased the PS of F5-LEF from 185.6 to 198.2 nm and slightly decreased the zeta potential value (Table 2).

### 3.2. Transmission Electron Microscopy

**Figure 2:**
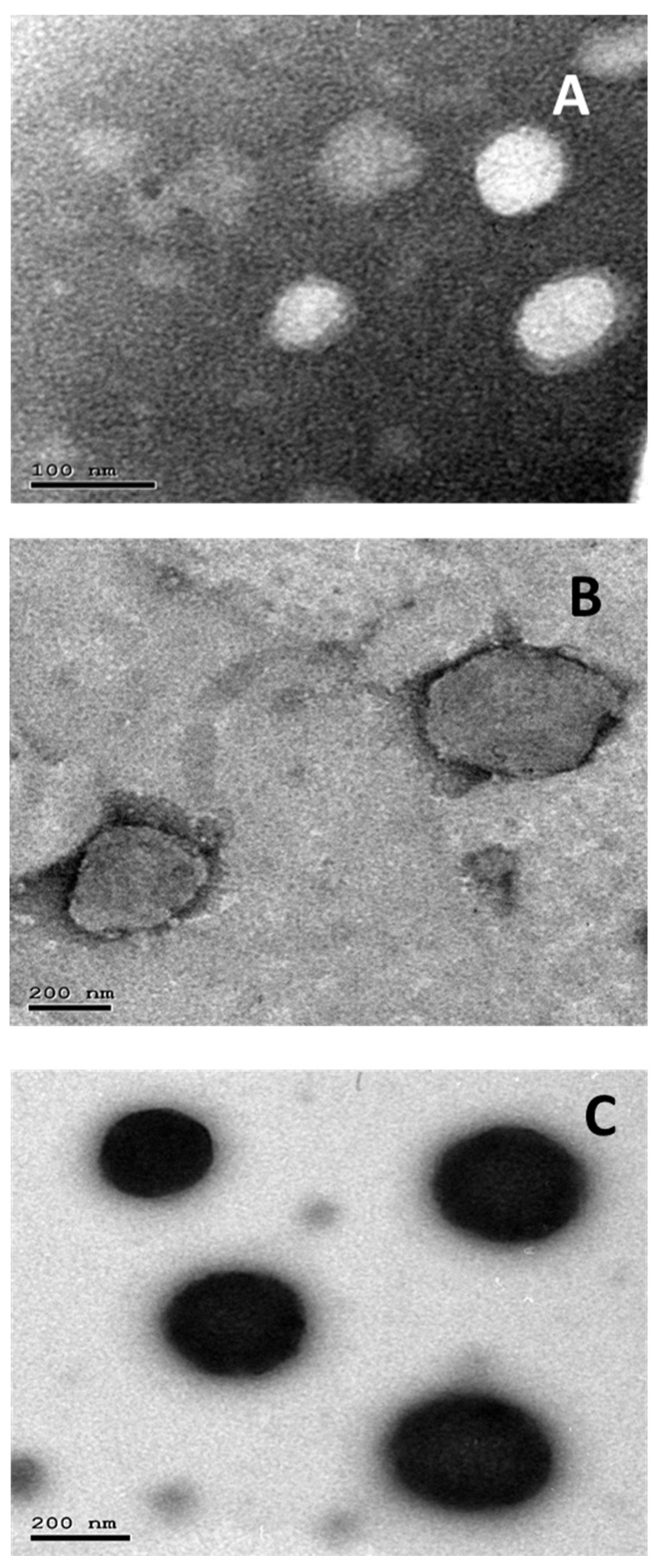
TEM micrographs of: (**A**) plain EMLs, (**B**) LEF loaded EMLs, and (**C**) LEF SPION EMLs.

TEM micrographs revealed that different EML formulations, i.e., blank, LEF EMLs, and LEF SPION EMLs, displayed a spherical shape with no signs of aggregation (Figure 2). In addition, the core-shell structure was observed in the blank and LEF EMLs (Figure 2A,B).

### 3.3. FTIR

**Figure 3:**
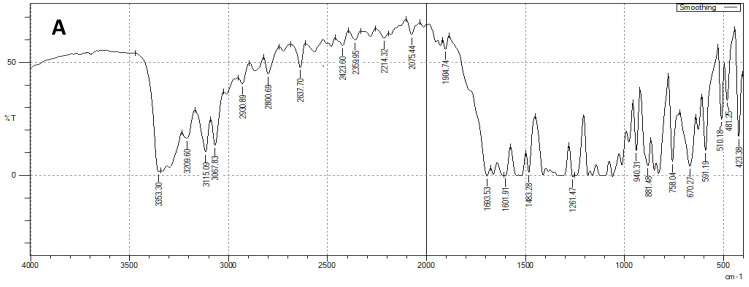

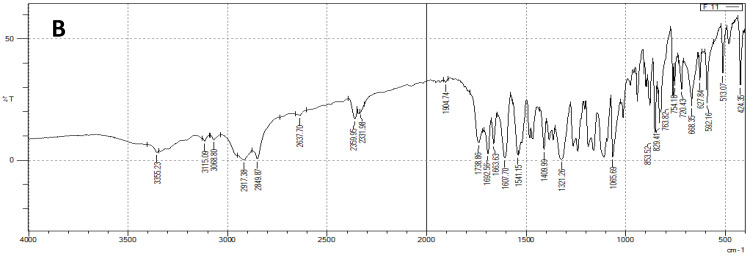

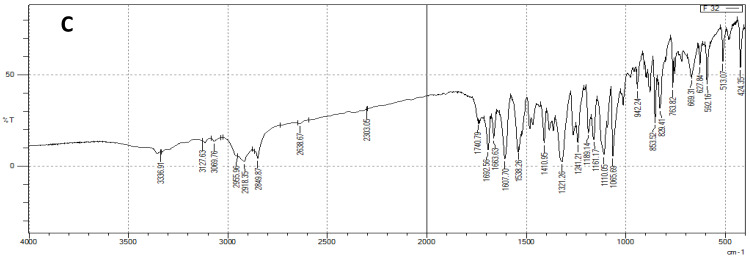
FTIR spectra of: (**A**) LEF, (**B**) LEF EMLs physical mixture, and (**C**) selected EMLs formulation.

Figure 3A illustrates the FTIR spectrum of LEF. It shows several characteristic peaks. Peaks at 1601 and 1540 cm^−1^ represent amide group peaks. The isooxsazole peak appeared at 1693 cm^−1^. Moreover, at 3067 and 2930 cm^−1^, the peaks of aromatic CH and CH stretching appeared, respectively. Compritol displayed characteristic peaks at 2849 and 1738 cm^−1^ that correspond to C-H and C-O stretching vibrations, respectively (Figure 3B). This is in agreement with previous reports [22,31]. Cholesterol displayed several characteristic peaks in both physical mixture and EMLs formulation. Peaks at 1321, 1663, and 2917 cm^−1^ represent CH_2_ bending, C=C and CH_2_, and CH_3_ asymmetric stretching vibrations, respectively. These findings are along with previously reported results [55]. Phosphatidyl choline peaks appeared at 1065, 1738, and 2917 cm^−1^ and represent P=O, C=O, and CH_2_ and CH_3_ asymmetric stretching vibrations, respectively [56]. LEF characteristic peaks and other components characteristic peaks appeared in EMLs physical mixture (Figure 3B) and selected EMLs formulation spectra (Figure 3C), indicating absence of interactions between formulation components and the drug.

### 3.4. DSC

**Figure 4:**
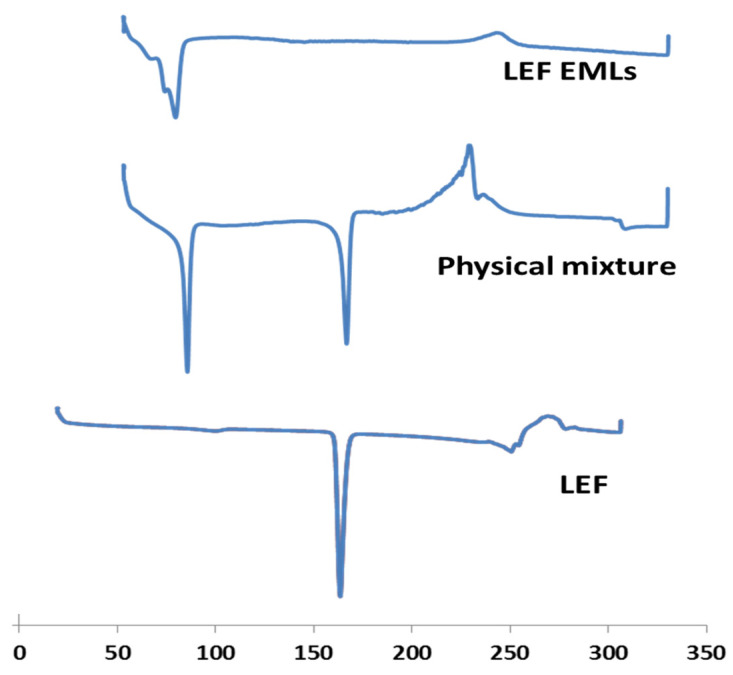
DSC spectra of LEF, LEF EMLs physical mixture, and selected EMLs formulation.

DSC is a valuable tool to study and examine melting and recrystallization behavior of different materials and lipids of interest here. LEF, physical mixture of all components, and selected LEF-EMLs formulation were analyzed by DSC. As Figure 4 illustrates, LEF thermogram showed sharp characteristic endothermic peak at 162 °C, while that of lipid mixture composed of Compritol, PC and cholesterol appeared at 73 °C. LEF peak disappeared in selected EMLs formulation spectrum indicating that LEF molecularly disappeared in the lipid matrix [22].

### 3.5. In Vitro Drug and Kinetics Release

A solubility study of LEF was conducted in PBS (pH 7.4), and it was found to be 2.67 mg/100 mL. The release volume was chosen to be 75 mL to ensure sink conditions [57].

**Figure 5:**
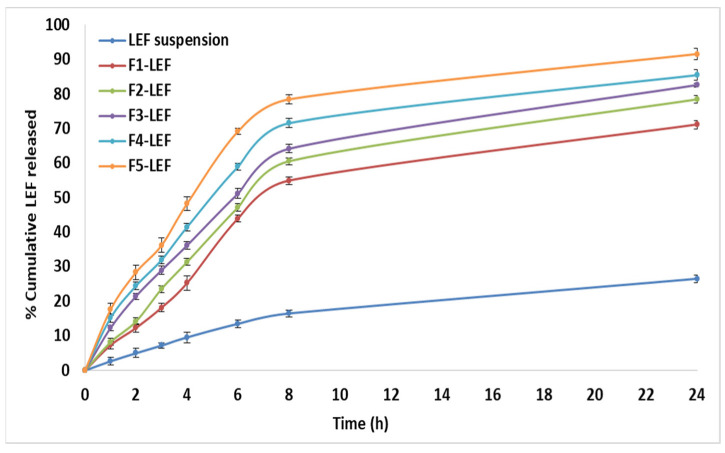
Cumulative percentage LEF released from LEF loaded EMLs.

Figure 5 shows that LEF release from different LEF-loaded EMLs had a two-phase release pattern. First, there was a burst effect in the first hour, and then, there was a steady release for 24 h. In the first hour, F1, F2, F3, F4, and F5 showed 7.3%, 7.8%, 12.3%, 15.2%, and 17.6% LEF release, respectively. This burst effect may be assigned to the release of LEF adsorbed on the EMLs’ surface and in the interphase within the layered phospholipids’ shell. Prolonged LEF release may be attributed to the extended time needed for LEF to diffuse from the EML matrix [12].

F1-LEF had the largest PS and slowest drug release during the entire experiment. On the other hand, F5-LEF had the smallest particle size and fastest drug release, likely because the increased particle size decreased the exposed surface area, slowing down the rate of drug release [10,52].

After fitting the aforementioned release data to zero, first order, Higuchi and Korsmeyer– Peppas equations, the coefficient of variation (R^2^) was recorded (Table 3). LEF release from suspension followed fitted first order release kinetics, upon its incorporation in EMLs. It followed the Higuchi model indicating a combination of diffusion and dissolution release mechanisms [31].

### 3.6. In Vivo Studies

Despite the fact that RA is a systemic disease, IA injections are given to individual inflamed joints that do not respond to systemic treatment [58,59,60]. Using the IA route, drugs can be delivered to the joint space, where they can be more effective and have fewer side effects [61]. In addition, IA route addresses the concerns about the extent of bioavailability and other drug changes that can decrease the efficacy of systemic drug delivery [31,58,62].

Several models of RA induction have been reported including collagen type II induced arthritis, adjuvant induced arthritis, and antigen induced arthritis [21]. 

In this study, RA was induced using an adjuvant-induced arthritis model that can effectively induce RA in different rat strains [31,63]. IA injections of CFA which is formed of mycobacterium suspended in mineral oil can cause RA [22]. The RA induction and treatment protocols were performed as previously reported [6,31,64]. Treatment was started on the third day of RA induction, and the administered dose of LEF was 10 mg/kg. During treatment with the LEF SPION EMLs, an external magnet was placed near the injected knee to maximize the localization of the injected EMLs in the joint tissues (Figure 1B). Butoescu et al. [54] outlined that the magnetic retention of dexamethasone-loaded PLGA microparticles was enhanced by extending the exposure time to magnetic field. They also reported a 10 min exposure was sufficient to ensure 100% retention of microparticles in the joints [54]. It was also reported that the encapsulation of SPIONs in dexamethasone loaded PLGA microparticles resulted in retention of the microparticles in the joint upon the application of an external magnet, thus possibly reducing their clearance from the joint. Dexamethasone release profiles were shown to be quite similar in vitro (6 days) and in vivo, using a mouse dorsal air pouch model (5 days) [65]. 

#### 3.6.1. Average Joint Diameter

**Figure 6:**
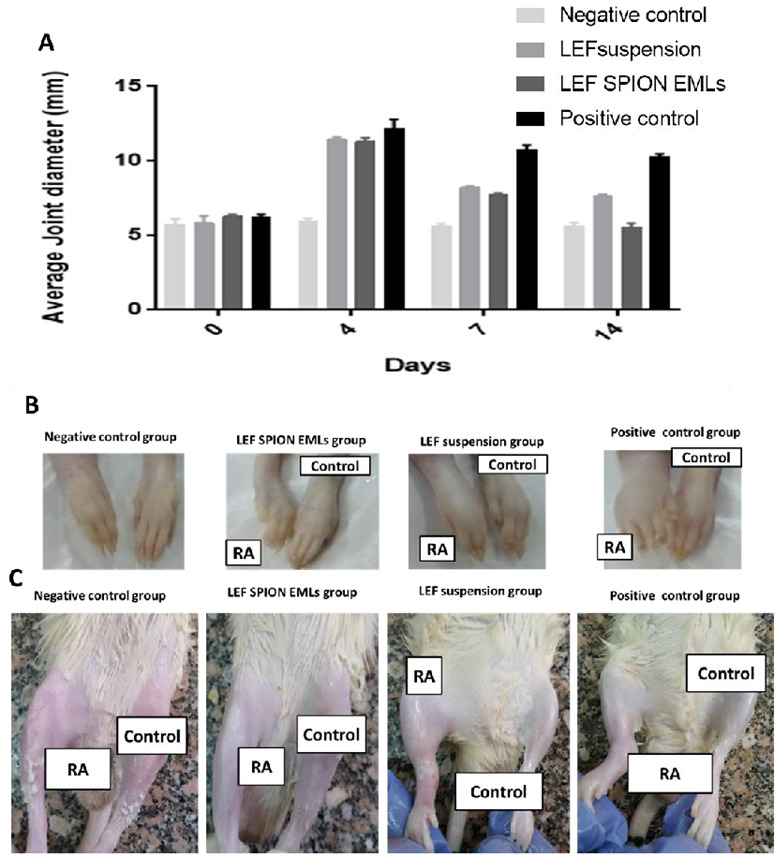
(**A**) Average joint diameter of different experimental groups at days 0, 3, 7, and 14. (**B**) and (**C**) are photographs of different groups of rat paws and joints 14 days post-treatment, respectively.

The average joint diameter of each group measured on days 0, 3, 7, and after 14 post treatment was shown in Figure 6A. Group treated with LEF SPION EMLs showed normal joint diameter after 14 days of treatment with statistically insignificant difference from the negative control (Two-way ANOVA, *p* < 0.001). Both LEF treated groups revealed significant improvement in joint diameter in comparison with the positive control group (two-way ANOVA, *p* < 0.001).

#### 3.6.2. X-ray Radiographic Examination

**Figure 7:**
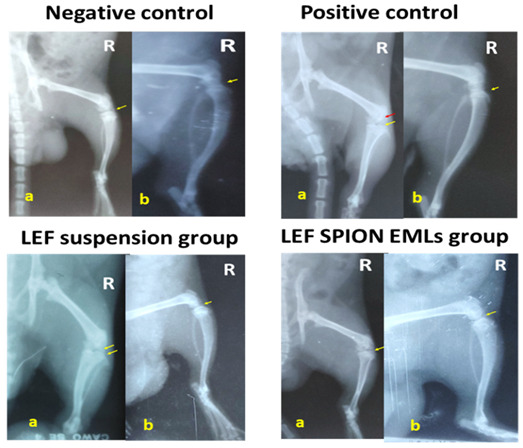
X-ray photographs of the right knee joint of different experimental groups, where (**a**) is the antero-posterior view and (**b**) is the latero-medial view.

Figure 7 illustrates different joint radiographic examination of knee joints in different groups. The negative control group exhibited normal joint soft tissue and space radiodensity and clear femoral condyle and proximal tibia (yellow arrow). The positive control group showed joint effusion with narrowing and increased joint space radiodensity. The femoral condyle and tibial showed some osteophytic reactivity (red and yellow arrows). The group treated with LEF SPION EMLs showed better joint healing compared to the group treated with LEF suspension as only slight joint effusion was recorded and the joint space almost clear with very low osteophytic reactivity within the joint. Meanwhile, the group treated with LEF suspension showed joint effusion and swelling with increased joint space radiodensity and osteophytic reactivity at both femoral condyle and proximal tibia (yellow arrow).

#### 3.6.3. RT-PCR Analysis of PERK, PJNK, and P 38 MAPK

**Figure 8:**
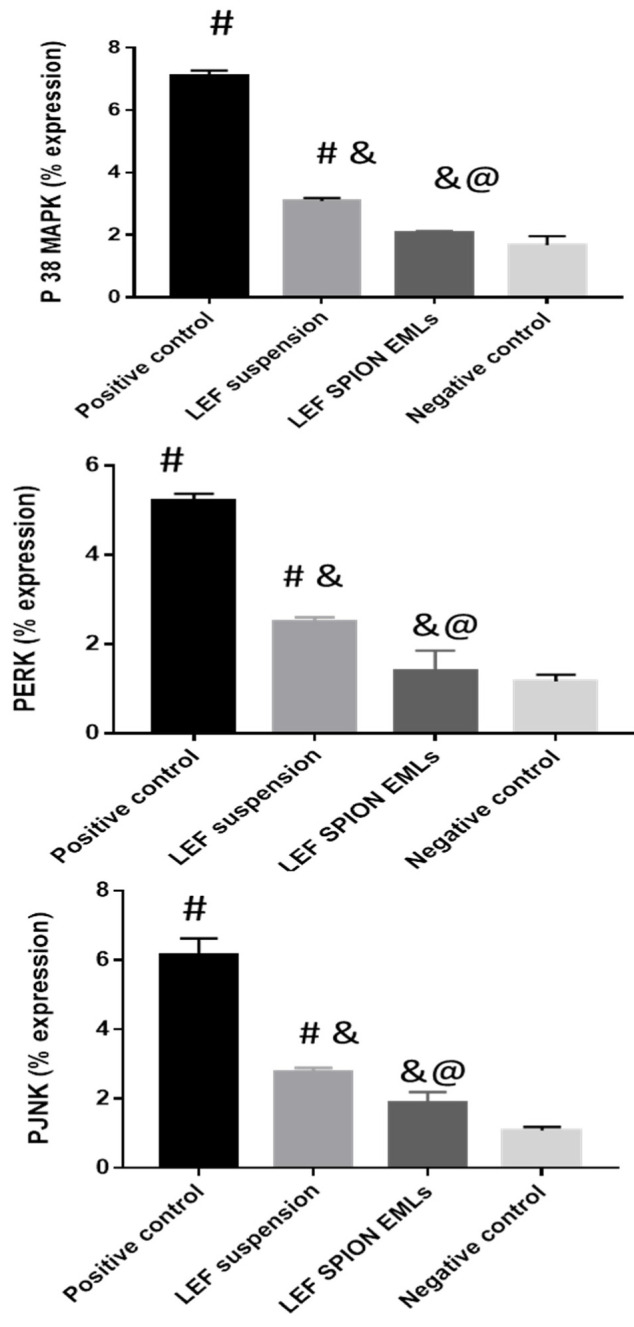
PERK 1/2, PJNK, and P38 MAPK levels assessed by PCR in dissected joints. Values are expressed as mean ± SD; n = 3. Statistical analyses were performed using one-way analysis of variance (ANOVA) followed by Tukey’s post hoc test. # significant as compared to negative control group. & significant as compared to positive control group. @ significant as compared to LEF suspension. Significant difference was conducted at *p* < 0.0001.

MAPKs are attractive targets for treating RA as they modulate cell proliferation, apoptosis, cytokine gene expression, and the production of metalloproteinase. In addition, the synthesis of pro-inflammatory cytokines is managed by the MAPK pathway [66]. The three major MAP kinase families, ERK, JNK, and p38, in the dissected joints of different experimental groups were assessed (Figure 8). The genes mentioned above are upregulated in patients with RA [67]. For example, p38 has an important role in cytokine production and cytokine-induced osteoclastogenesis [68]. In addition, PJNK signaling drives collagenase-3 gene expression and bone damage in rats with adjuvant-induced arthritis [67].

Results revealed that the positive control group had significantly higher levels of PERK 1/2, PJNK, and P38 MAPK genes than the other groups. Both LEF suspension and LEF SPION EMLs groups showed lower gene expression level in comparison to the positive control group. Gene levels in the group treated with LEF SPION EMLs did not show any significant differences from the negative control group.

#### 3.6.4. Western Blot Analysis

Blood samples were tested for Bcl-2, Bax, and cleaved caspase levels using Western Blotting. Mitochondrial homeostasis and cell viability are dependent on the expression of Bcl-2 genes in synovial fibroblasts. The Bcl-2 gene family encounters several proteins that control mammalian cells apoptosis. For example, Bcl-2, Mcl-1, and Bcl-XL inhibit cell apoptosis, whereas Bax, Bad, Bik, Bak, and Bcl-XS promote apoptosis [1]. In addition, the imbalance between Bax and Bcl-2 affects the susceptibility of chondrocytes to apoptosis. Furthermore, caspase-3 mediates apoptosis process in chondrocytes [32]. In patients with RA, the expression of the pro-apoptotic Bax gene is reduced while that of the anti-apoptotic Bcl-2 gene is enhanced [69].

**Figure 9:**
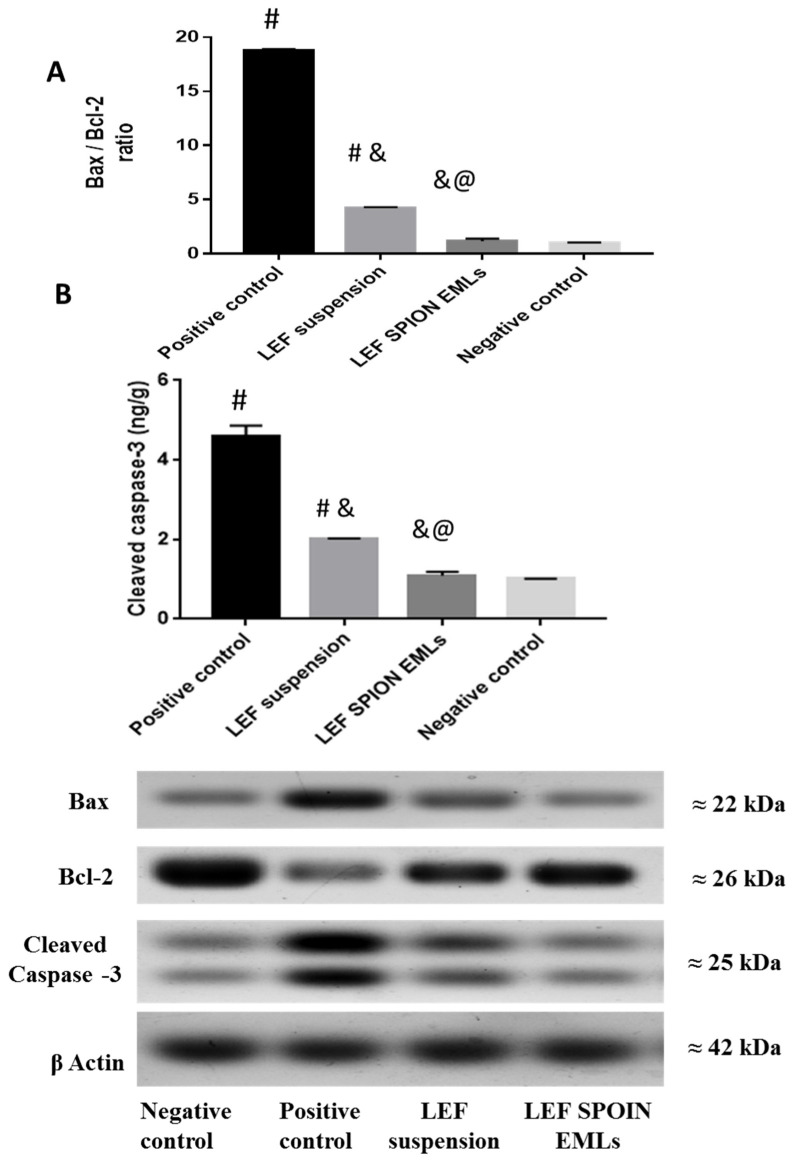
Effect of LEF suspension and LEF SPION EMLs on Bax and Bcl-2 ratio (**A**) and cleaved caspase-3 level (**B**) in joints of different experimental groups. Values are expressed as mean ± SD; n = 3. Statistical analyses were performed using one-way analysis of variance (ANOVA) followed by Tukey’s post hoc test. # significant as compared to negative control group. & significant as compared to positive control group. @ significant as compared to LEF suspension. Significant difference was conducted at *p* < 0.0001.

Analysis of the aforementioned proteins revealed that the positive control group had the highest Bax/Bcl-2 ratio (Figure 9A) and cleaved caspase-3 (Figure 9B). The groups treated with the LEF suspension and LEF SPION EMLs demonstrated decreased Bax/Bcl2 ratio and cleaved caspase-3 levels. Furthermore, the group treated with LEF SPION EMLs exhibited comparable protein levels to the negative control group.

#### 3.6.5. Enzyme-Linked Immunosorbent Assay (ELISA)

Several factors can cause RA progression, including inflammatory cascades, high levels of pro-inflammatory cytokines like TNF-α and IL-1b [6,70], and low levels of NRF2 factors [71]. Pro-inflammatory cytokines can stimulate articular chondrocytes and synoviocytes to synthesize matrix-degrading and pro-inflammatory enzymes, eventually increasing cytokine production, inflammation, and cartilage degeneration [72,73].

RA progression is largely influenced by TNF-α, which plays a key role in regulating other inflammatory mediators [74,75,76]. It has been reported that IL-1, IL-6, and IL-8 production was reduced in cultures of synovial cells from patients with RA when antibodies were used to block TNF-α signaling [21,75]. Moreover, MMP-9 suppresses RA progression by reducing synovial fibroblast proliferation via the decreased synthesis of IL-1β, IL-6, IL-8, and TNF-α [77]. In addition, NRF2 can bind effectively to antioxidant response elements, exerting a cellular defensive mechanism to remove cytotoxic electrophiles and reactive oxygen species and protect the tissues [71]. Furthermore, SOD plays an important role in scavenging free radicals, reducing inflammation, and relieving joint pain [78].

**Figure 10:**
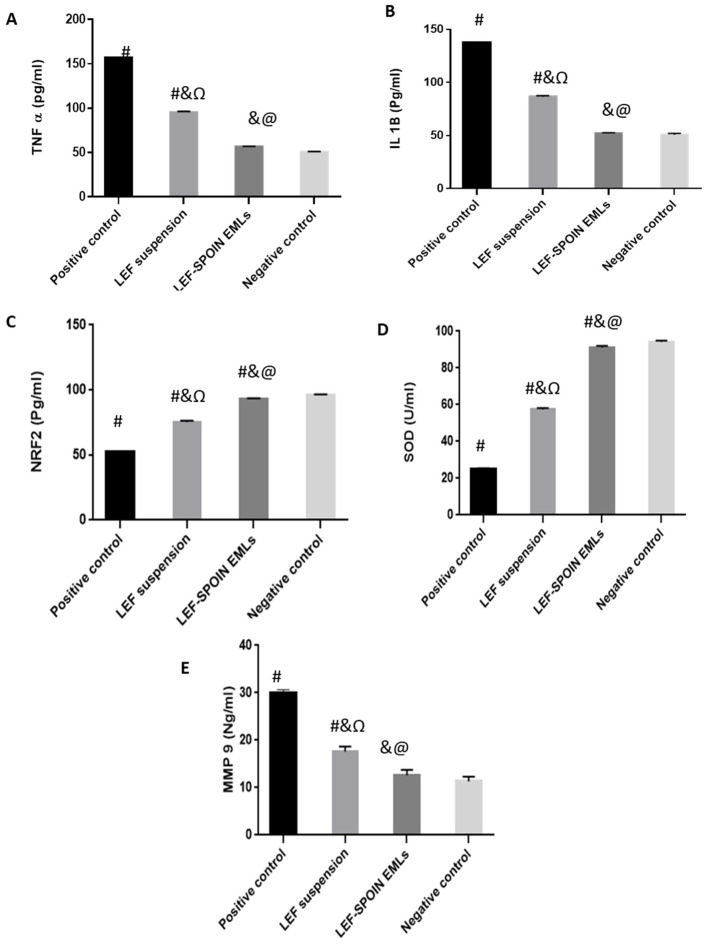
Level of different biomarkers at the end of the experiment: (**A**) TNFα level, (**B**) IL1β level, (**C**) NRF 2 level, (**D**) SOD level, and (**E**) MMP 9 level. Statistical analyses were performed using one-way analysis of variance (ANOVA) followed by Tukey’s post hoc test. # significant as compared to negative control group. & significant as compared to positive control group. @ significant as compared to LEF suspension. Ω significant as compared to LEF-SPION EMLs. Significant difference was conducted by one-way ANOVA at *p* < 0.0001.

In this study, the levels TNF-α, IL1B, and MMP-9, usually elevated in patients with RA, were analyzed [6,70,79,80]. In addition, NRF2 and SOD, usually present at low levels in rats with RA, were evaluated [71,78]. The levels of TNF-α, ILB 2, and MMP-9 were significantly decreased in the groups treated with LEF suspension and LEF SPION EMLs compared to the positive control (Figure 10). Meanwhile, the levels of the cytokines mentioned above were more reduced in the LEF SPION treatment groups than in that with LEF suspension. For example, IL1B levels were 137, 86.5, 52, and 50.5 pg/mL for positive control, group treated with LEF suspension, group treated with LEF-SPION EMLs, and negative control, respectively. On the other hand, levels of NRF2 and SOD significantly increased compared to the positive control group. NRF 2 levels were 52.1, 75.8, 93.2, and 96 pg/mL for positive control, group treated with LEF suspension, group treated with LEF-SPION EMLs, and negative control, respectively.

One way ANOVA (*p* < 0.0001) revealed a statistically significant difference between the groups treated with LEF SPION EMLs and LEF suspension and the positive group, while the group treated with LEF SPION EMLs was statistically not significantly different from the negative control group.

As previously reported by RT-PCR results, inhibition of pro-inflammatory cytokine production and prevention of osteoclast formation through blockade of the MAPK signaling pathway may prevent inflammatory bone loss at multiple levels [68].

#### 3.6.6. Histopathologic Studies & Immunohistochemical Staining

**Figure 11:**
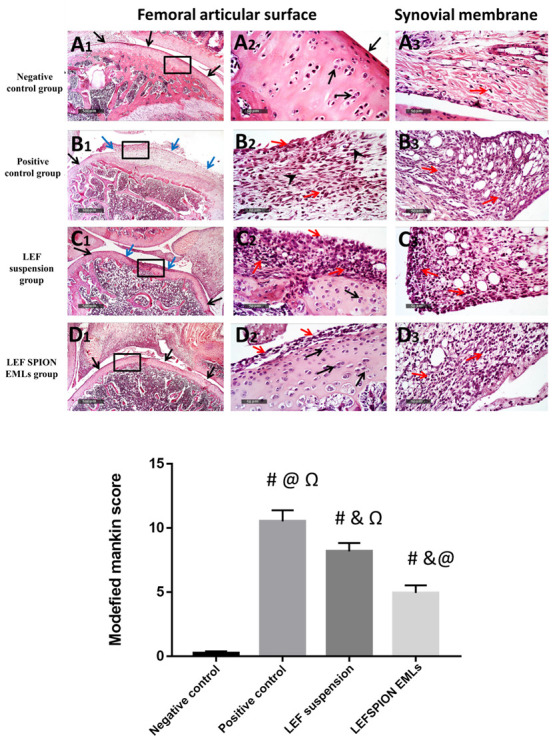
Morphological features of articular surfaces and synovial membranes from different groups: (**A1**–**A3**) negative control group, (**B1**–**B3**) positive control group, (**C1**–**C3**) LEF suspension group, and (**D1**–**D3**) LEF SPION EMLs group. H&E stain 40× & 400×. Black arrow = apparent intact cartilage, blue arrow = damaged cartilaginous surfaces, red arrow = inflammatory cells infiltrates, arrow head = fibroblastic proliferation. Values are expressed as mean ± SD; n = 6. Statistical analyses were performed using one-way analysis of variance (ANOVA) followed by Tukey’s post hoc test. # significant as compared to negative control group. & significant as compared to positive control group. @ significant as compared to LEF suspension. Ω significant as compared to LEF-SPION EMLs. Significant difference was conducted by one-way ANOVA at *p* < 0.0001.

**Figure 12:**
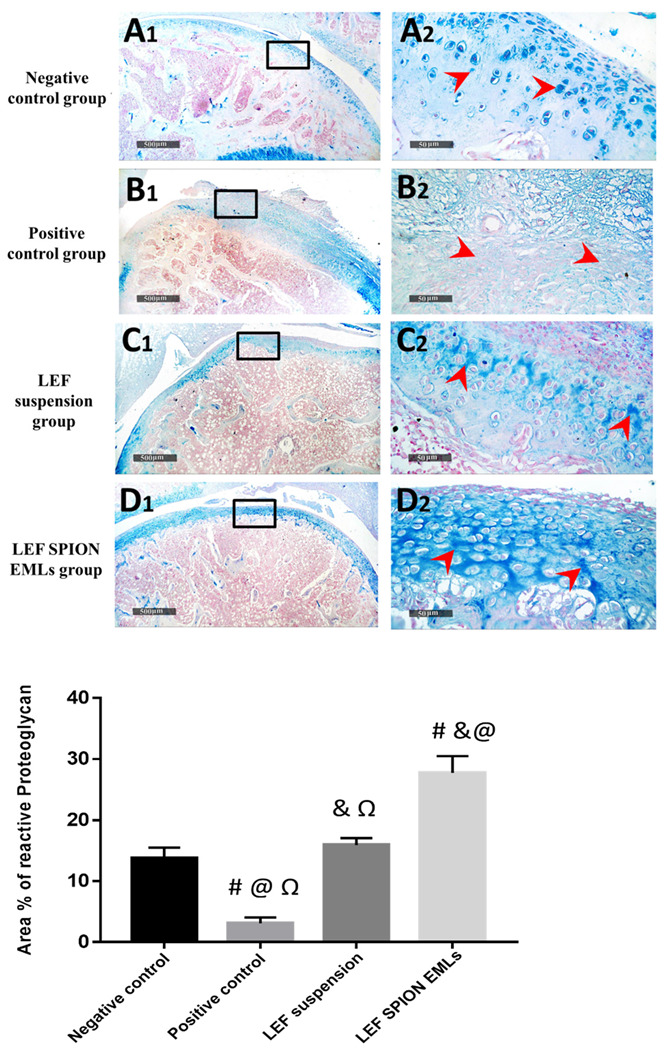
Demonstrating articular cartilage proteoglycans staining affinity to alcian blue staining in different groups: (**A1**,**A2**) negative control group, (**B1**,**B2**) positive control group, (**C1**,**C2**) LEF suspension group, and (**D1**,**D2**) LEF SPION EMLs group 400×. Values are expressed as mean ± SD; n = 6 (Microscopic field = 78,489 μm^2^). Statistical analyses were performed using one-way analysis of variance (ANOVA) followed by Tukey’s post hoc test. # significant as compared to negative control group. & significant as compared to positive control group. @ significant as compared to LEF suspension. Ω significant as compared to LEF-SPION EMLs. Significant difference was conducted by one-way ANOVA at *p* < 0.0001.

Microscopic examination of femoral articular surface of knee joints revealed that the control samples demonstrated normal organized histological features of articular surfaces with apparent intact hyaline cartilage with regular smooth surface and many apparent intact chondrocytes were observed in lacunae with large vesicular nuclei (Figure 11A1,A2—arrows) at different zones with up to 13.7% of relative cartilaginous matrix and peri-lacunar areas showed positive proteoglycans reactivity for alcian blue stain (Figure 12A1,A2). Normal histological features of synovial membranes were shown with minimal inflammatory cells infiltrates (Figure 11A3—arrows) and abundant sub-epithelial collagen fibers with normal vasculatures. Model samples showed wide areas of cartilaginous surface erosions and disruption (Figure 11B1,B2) with marked loss of normal organization of chondrocytes at different cartilaginous zones and replaced with fibrous tissue with significant fibroblastic activity (Figure 11B2 arrow head) mixed with abundant mononuclear cells infiltrates (Figure 11B2 red arrow). In addition to Sever inflammatory cells infiltrates (Figure 11B3 red arrow) as well as hyperplasia of synovial membranes were shown. The mean modified Mankin scoring system for model osteoarthritis samples was up to 10 ± 0.8. Marked loss and decrease of cartilaginous matrix proteoglycans to alcian blue staining (up to 3.1% of relative matrix area percentage) (Figure 12B1,B2). T1 samples group showed mild better protective efficacy of articular cartilage with persistent focal areas of degenerative changes of superficial cartilaginous layers with higher reactive inflammatory cells infiltrates as well as fibroblastic activity (Figure 11C1,C2) with apparent intact deeper cartilaginous zones. Moreover; persistent abnormal inflammatory cells infiltrates were shown in synovial membranes of different sample (Figure 11C3). The mean modified Mankin scoring system for T1 samples was up to 8.16 ± 0.65. Moreover; up to 5 folds increase of area percentage of reactive matrix proteoglycans density to alcian blue staining was recorded (Figure 12C1,C2). Histological examination of T2 samples showed more enhanced protective efficacy and histological features of different joints. Mild focal areas of articular surface irregularities with fibroblastic activity and minor inflammatory cells infiltrates were shown (Figure 11D2 red arrows). However; moderate persistent inflammatory reaction in synovial membranes (Figure 11D3 red arrows). The mean modified Mankin scoring system was up to 4.9 ± 0.14. Moreover; up to 8.9 folds increase of reactive matrix proteoglycans to alcian blue staining compared with model samples (Figure 12D1,D2).

**Figure 13:**
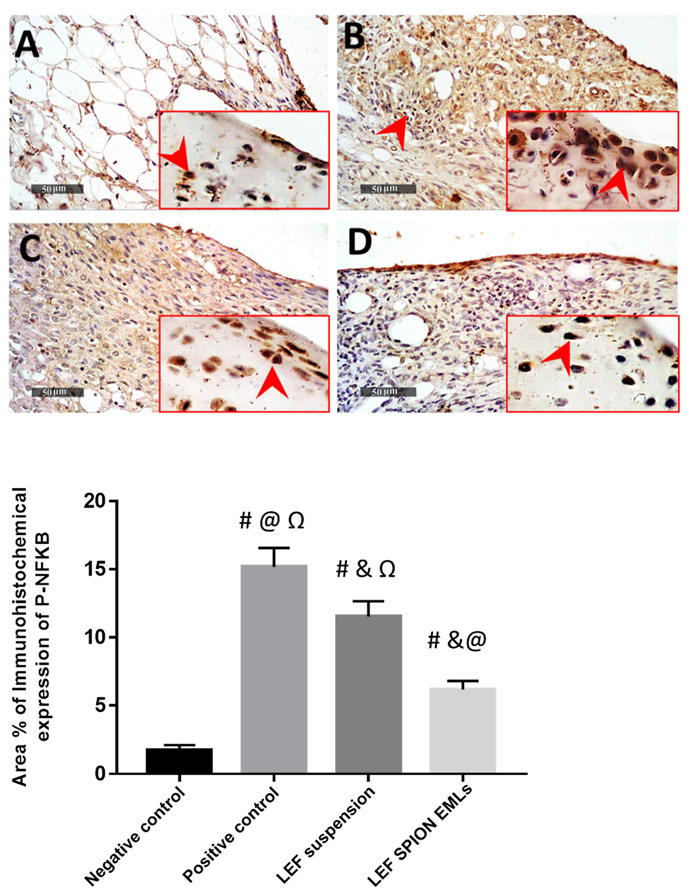
P-NFkB Immunohistochemical expression levels in articular surface chondrocytes and synovial membranes of different groups: (**A**) negative control group, (**B**) positive control group, (**C**) LEF suspension group, and (**D**) LEF SPION EMLs group 400×. Values are expressed as mean ± SD; n = 6. Statistical analyses were performed using one-way analysis of variance (ANOVA) followed by Tukey’s post hoc test. # significant as compared to negative control group. & significant as compared to positive control group. @ significant as compared to LEF suspension. Ω significant as compared to LEF-SPION EMLs. Significant difference was conducted by one-way ANOVA at *p* < 0.0001.

Immunohistochemical analysis of phospho NFkB expression levels in articular surfaces chondrocytes and synovial membranes revealed; significant up to 8.8 folds increase of mean expression levels in Model samples compared with normal controls. However; significant reduction of mean expression levels in T1 and T2 samples up to 23.8% and 59.6% respectively compared with Model osteoarthritis samples, (Figure 13).

## 4. Conclusions

Leflunomide loaded bioemulsomes (LEF EMLs) were successfully prepared using the thin-film hydration method and optimized by varying homogenization parameters (speed and time) and drug loading. F5 showed the highest %EE of the drug, and a favorable size and fastest drug release was selected to load SPION to enhance drug retention using an external magnetic field. TEM micrographs of the selected formulation showed spherical particles with no aggregations. The in vivo results demonstrated the ability of the LEF suspension and LEF SPION EMLs to impede rheumatoid arthritis progression with a superiority of the LEF SPION EMLs over LEF suspension. Therefore, LEF SPION EMLs could be considered as an efficient platform for the suppression of RA.

## Figures and Tables

**Table 1:** The primer sequence of genes selected for polymerase chain analysis.

Gene	Forward Primer	Reverse Primer
β-Actin	5′-TATCCTGGCCTCACTGTCCA-3′	5′-AACGCAGCTCAGTAACAGTC-3′
PERK 1/2	5′-TCAAGCCTTCCAACCTC-3′	5′-GCAGCCCACAGACCAAA-3′
PJNK	5′-GAAGAGTAGCAAGACAGGGA-3′	5′- GAAGAGGCGGTCAAAGGA-3′
p38-MAPK	5′-AGGGCGATGTGACGTTT-3′	5′-CTGGCAGGGTGAAGTTGG-3′

**Table 2:** Optimization and colloidal characteristics of blank, LEF EMLs and LEF-SPION EMLs using high-shear homogenization.

Formulation Code	Homogenization		Particle Size (nm) ± SD	PDI ± SD	Zeta Potential (mV) ± SD	EE % ± SD
	Speed (rpm)	Time (min)				
F1	10,000	0	416.12 ± 2.3	0.256 ± 0.0014	−23.5 ± 1.3	-----
F1-LEF			436.2 ± 1.06	0.112 ± 0.004	−31.5 ± 1.25	74.2 ± 0.85
F2		5	398.25 ± 1.45	0.15 ± 0.001	−25.3 ± 2.03	-----
F2-LEF			425.6 ± 1.8	0.14 ± 0.0012	−32.6 ± 2.1	75.2 ± 1.04
F3		10	365.3 ± 1.47	0.142 ± 0.003	−22.3 ± 0.98	
F3-LEF			385.4± 1.4	0.151 ± 0.005	−24.6 ± 2.05	76.4 ± 1.04
F4		15	223.6 ± 1.08	0.014 ± 0.012	−28.3 ± 2.1	-----
F4-LEF			245.3 ± 1.12	0.005 ± 0.014	−31.2 ± 2.23	81.5 ± 1.74
F5-LEF	15,000	5	178.6 ± 1.2	0.008 ± 0.0012	−22.3 ± 1.14	-----
F5			185.6 ± 0.87	0.004 ± 0.004	−25.6 ± 0.65	87.3 ± 1.2
LEF-SPION F5			198.2 ± 1.8	0.004 ± 0.001	−20.5 ± 0.85	86.4 ± 1.05
SPION			10.2 ± 2.05	0.16 ± 0.0 3	23.2 ± 1.22	-----

**Table 3:** Release kinetics.

Model	R^2^					
	LEF Suspension	F1	F2	F3	F4	F5
Zero order	0.885	0.783	0.783	0.771	0.71	0.668
First order	**0.989**	0.705	0.960	0.946	0.911	0.985
Higuchi	0.909	**0.929**	**0.966**	**0.973**	**0.973**	**0.996**
Korsmeyer–Peppas	0.976	0.921	0.921	0.933	0.92	0.908

## Data Availability

All data are reported in the manuscript.
